# Dual-Dewetting Process for Self-Assembled Nanoparticle Clusters in Wafer Scale

**DOI:** 10.3390/ijms241713102

**Published:** 2023-08-23

**Authors:** Minjun Kim, Hyun-Ju Ahn, Vanna Chrismas Silalahi, Damun Heo, Samir Adhikari, Yudong Jang, Jongmin Lee, Donghan Lee

**Affiliations:** 1 Department of Physics, Chungnam National University, Daejeon 34134, Republic of Korea; 2 School of Semiconductor Display Technology, Hallym University, Chuncheon 24252, Republic of Korea; 3 Institute of Quantum Systems (IQS), Chungnam National University, Daejeon 34134, Republic of Korea; 4 Nano Convergence Technology Center, Hallym University, Chuncheon 24252, Republic of Korea

**Keywords:** plasmonic molecules, self-assembled nanoparticle, dewetting precess, electric field enhancement

## Abstract

Plasmonic molecules, which are geometrically well-defined plasmonic metal nanoparticle clusters, have attracted significant attention due to their enhancement of light–matter interactions owing to a stronger electric field enhancement than that by single particles. High-resolution lithography techniques provide precise positioning of plasmonic nanoparticles, but their fabrication costs are excessively high. In this study, we propose a lithography-free, self-assembly fabrication method, termed the dual-dewetting process, which allows the control of the size and density of gold nanoparticles. This process involves depositing a gold thin film on a substrate and inducing dewetting through thermal annealing, followed by a second deposition and annealing. The method achieves a uniform distribution of particle size and density, along with increased particle density, across a 6-inch wafer. The superiority of the method is confirmed by a 30-fold increase in the signal intensity of surface-enhanced Raman scattering following the additional dewetting with an 8 nm film, compared to single dewetting alone. Our findings indicate that the dual-dewetting method provides a simple and efficient approach to enable a variety of plasmonic applications through efficient plasmonic molecule large-area fabrication.

## 1. Introduction

Plasmonics has attracted much attention in optics, thermo-optics, and electro-optics due to the strong enhancement of electric fields and light–matter interactions by surface plasmons. We call collections of particles with well-defined strong surface plasmon resonance on the surface of metal nanoparticles plasmonic molecules [1,2]. The properties of plasmonic molecules offer advantages in diverse applications such as photovoltaics [3,4,5], fluorescence sensing [6], and surface-enhanced Raman scattering (SERS) [7,8,9,10,11,12,13,14,15,16]. Moreover, plasmonic molecules have enabled unprecedented sensitivity and specificity in scientific fields such as biomedical diagnostics [13] and environmental monitoring [17]. Manipulating the size, shape, and composition of plasmonic molecules provides numerous opportunities for applications in various fields [2].

The properties of plasmonic molecules are sensitively affected by their geometry. For this reason, researchers have explored various approaches to control the size and density of plasmonic nanostructures, including chemical synthesis [18,19,20,21], lithography [14,15,22], and dewetting [3,23,24,25,26,27] to fabricate plasmonic molecules. Metal nanoparticles synthesized in a solution have high reproducibility and homogeneity. However, chemical reactions or other interactions with the substrate are needed to align the nanoparticles. Furthermore, lithography-based processes, which can be used to draw precise structures on substrates, face problems in low-cost, large-area fabrication. On the other hand, the dewetting method can generate nanoparticles in a large area by heat-treating thin metal films. The size and distribution of nanoparticles can be controlled by varying the thickness of the thin film as well as the temperature and time of the heat treatment. Despite these advantages, it has remained very difficult to simultaneously satisfy the size distribution and density of self-assembled nanoparticles.

**Figure 1:**
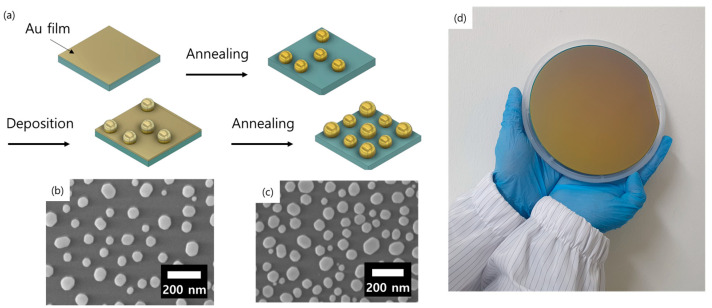
(**a**) Schematic diagram of the dual-dewetting process. (**b**,**c**) SEM images of gold nanoparticles immediately after the (**b**) first and (**c**) second dewetting. (**d**) Optical image of a 6-inch Si wafer with gold nanoparticles produced through the dual-dewetting process.

Performing dewetting multiple times is known as one of the methods to increase the number of particles and control the density and distribution [28,29,30,31]. In this study, we propose a method for precisely controlling the size and density of gold nanoparticles using a dual-dewetting process that precisely controls the thickness of the thin film. This process involves depositing a gold thin film on a substrate and inducing dewetting through heating the substrate, followed by a second deposition and thermal treatment. By repeating this deposition–annealing cycle, we can increase the number of gold nanoparticles such that the surface coverage and number of hotspots also increase. Figure 1a shows the fabrication flow. Additionally, we can achieve uniform particle distribution with no variation between the edge and center of the substrate at the 6-inch wafer level, as shown in the optical and scanning electron microscopy (SEM) images in Figure 1b,c. Our method is a time- and cost-efficient way to improve plasmonic properties by manipulating the density and size distribution of plasmonic molecules over a large area with neither lithography nor chemical synthesis.

## 2. Results and Discussion

### 2.1. Subsection Shape and Distribution Characteristics of Gold Nanoparticles by Dewetting

**Figure 2:**
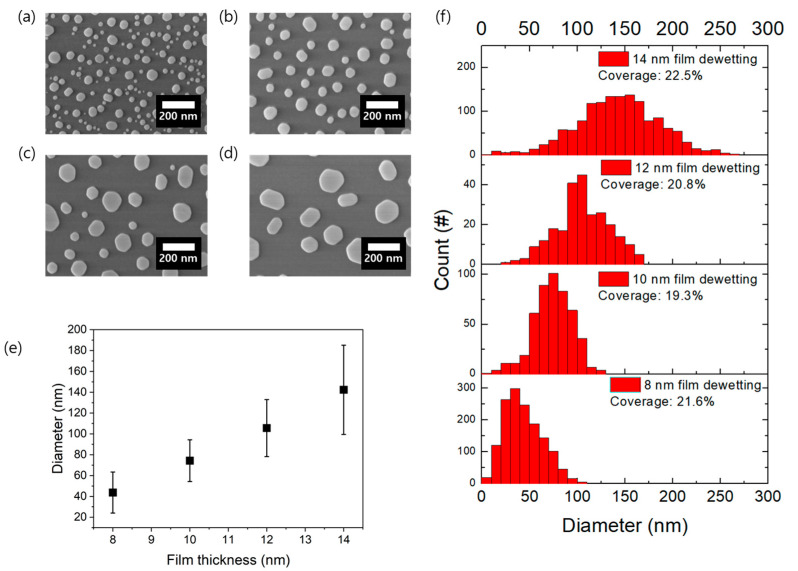
(**a**–**d**) SEM images of gold nanoparticles after a single dewetting with different thicknesses of gold thin films: (**a**) 8 nm, (**b**) 10 nm, (**c**) 12 nm, and (**d**) 14 nm. (**e**) Particle size as a function of film thickness. (**f**) Distribution of particle diameter and coverage. For more information, please refer to Supplement Figures S1 and S2 and Table S1.

We first performed a single dewetting to create gold nanoparticles. In our approach, using an E-beam evaporator, we deposited a gold film on a 300 nm SiO_2_ layer that was deposited by plasma-enhanced chemical vapor deposition (PECVD) on top of a Si substrate. The thickness of the gold film varied from 8 nm to 14 nm in increments of 2 nm. After annealing at 820 °C for 2 min, we observed the resulting nanoparticles using SEM, as can be seen in Figure 2a–d. From the images, we observed that the thicker the gold film, the larger the particle size. The particle size and distribution are shown in Figure 2e,f.

The average diameter of the particles was obtained from SEM images within an area of about 13 µm^2^. As shown in Figure 2e, film thicknesses of 8 nm, 10 nm, 12 nm, and 14 nm give an average diameter of 43.7 nm, 74.3 nm, 105.6 nm, and 142.3 nm, respectively. The result of this process shows a linear relationship between the size of the particles and the thickness of the thin film. This demonstrated that the size of the particles could be precisely controlled by adjusting the film thickness.

The augmentation of polydispersity in correlation with film thickness can be attributed to insufficient dewetting temperature. In experiments utilizing 8 nm films for dewetting (Supplement Figure S3), the variations in the dimensions of the resultant nanoparticles are minor, even when subject to alterations in the dewetting temperature. Conversely, an examination of 14 nm films reveals a significant increase in these variations (Supplement Figure S3). Furthermore, when the dewetting process is conducted at a temperature of 740 °C, the formation of nanoparticles occurs stably within a 7 nm film, whereas it is unsuccessful in a 14 nm film (Supplement Figure S4). This phenomenon can be elucidated by the increased energy requisites for deformation in the thicker film.

The particle distributions follow a Gaussian-like pattern, as shown in Figure 2f. Despite the increased particle size, we observed that the areal coverages of the gold nanoparticles are similar, around 20% in all cases. It is interpreted that the interparticle distance becomes larger as the size of the particles increases, which is confirmed from the SEM images. This means that there are almost no plasmonic molecules at a close distance between particles, thus indicating that it is difficult to fabricate a substrate with strong electric field enhancement with a single dewetting.

**Figure 3:**
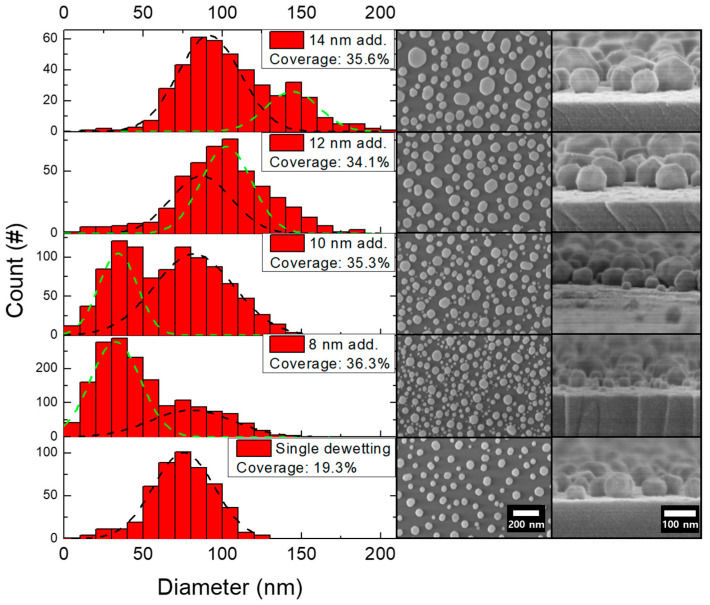
Size distribution along with top- and oblique-view SEM images of dual-dewetted gold nanoparticles with additional 8, 10, 12, and 14 nm films deposited on a single dewetting substrate. The single dewetting substrate was thermally annealed with a 10 nm gold thin film. The Gaussian fitting parameters are given in the Supplement Table S2. See Supplement Figure S5 for images of more locations.

To increase the particle coverage and density, we performed a second dewetting on the substrates that had previously undergone one dewetting. The 10 nm film was identified as the optimal source for the production of gold nanoparticles with diameters within the 80–100 nm range, making it suitable for incorporation into a SERS system. Specifically, we utilized gold particles made from a 10 nm film as the initial bare particle substrate and deposited additional 8, 10, 12, and 14 nm gold films on top of the substrates. After heat treatment at 820 °C for 2 min, we obtained SEM images as shown in Figure 3.

In all cases, the particle coverage increased to approximately 35%, indicating a 1.75-fold increase in coverage compared to the single dewetting case. The distribution of particles, however, was different from that of the single dewetted particles, which had a single Gaussian-like distribution. Instead, the distribution of the dual-dewetted particles appeared as two Gaussian profiles, which we attribute to two different particle types (See Supplement Figure S6 for simple mean particle size.): existing particles whose size increased due to the additional gold film deposition on top of the previously formed particles, and new particles derived from the newly deposited film only (similar to those in Figure 1a). To investigate the particle size distribution more precisely, we fit two Gaussian curves to the data. The average size of the particles formed in the first dewetting (black dashed line in Figure 3) was slightly increased through the second dewetting, while the new Gaussian curve (green dashed line) had a distinct average value. Notably, the average values of the diameter of the particles formed in the first dewetting after the second dewetting for the various gold film thicknesses were 80.6 nm for 8 nm, 82.1 nm for 10 nm, 86.7 nm for 12 nm, and 91.7 nm for 14 nm. Meanwhile, the green dashed line showed average values of 32.5 nm for 8 nm, 34.1 nm for 10 nm, 102.4 nm for 12 nm, and 144 nm for 14 nm. These values are similar to the average particle diameter for each film thickness that appeared after the first deposition. Such findings demonstrate that performing the dewetting process twice results in a much larger number of plasmonic molecules, giving a 1.75-fold increase in substrate coverage.

Furthermore, by controlling the thickness of the second film, we were able to adjust the size of the particles with additional density. Analogous to the single dewetting procedure, the double dewetting process results in thinner gold films generating a larger quantum of nanoparticles. Given the direct proportionality between the count of hotspots and that of nanoparticles, it would be reasonable to anticipate that a sample comprising thinner films would be more advantageous. Depending on the requirements, particles can be produced in various size combinations and in significantly larger quantities with a specific distribution.

### 2.2. Enhancement of Dual-Dewetted Gold Nanoparticles Tics of Gold Nanoparticles by Dewetting

**Figure 4:**
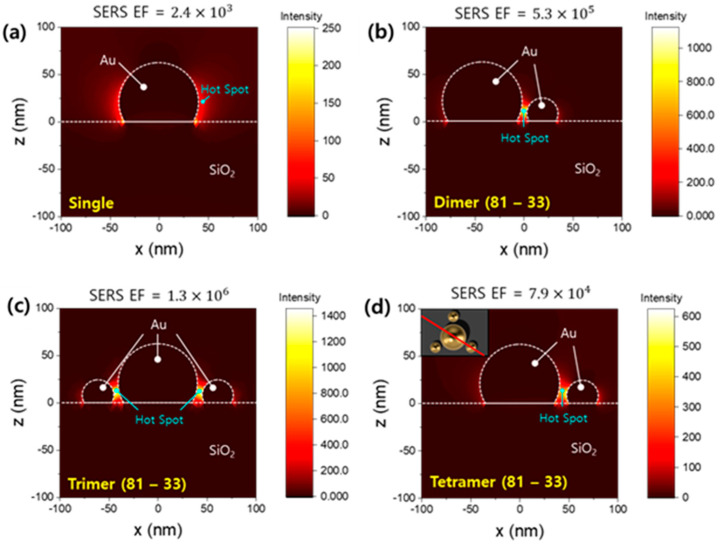
(**a**–**d**) Intensity distributions of a (**a**) single particle, (**b**) dimer with 81 nm and 33 nm particles, (**c**) trimer with a combination of 81 nm (center) and 33 nm (sides) particles, and (**d**) tetramer with a 81 nm main particle surrounded by three 33 nm particles. All particles are truncated by 25% of their diameter from the bottom. In the calculations, all particles are on a Si substrate having a 300 nm SiO_2_ layer on top. All gap sizes in (**b**–**d**) are 5 nm. The SERS enhancement factor was extracted at the indicated spot in each panel (cyan). While the (**a**–**c**) panels show the XZ plane, the data in (**d**) is shown in the diagonal plane corresponding to the red line in the inset.

We then conducted a numerical simulation to investigate the extent of the electric field enhancement for plasmonic molecules made up of different sizes compared to that for single particles [32,33,34]. For simplicity, x-polarized 633 nm light is incident on a single plasmonic molecule having a 5 nm gap between particles instead of a periodic structure so that we can eliminate coupling effects with the nearest particles in the simulation. Figure 4 shows light intensity distributions of different sets of gold particles at the XZ plane (Figure 4a–c) and the symmetric plane (Figure 4d). Figure 4a shows the result of a single particle. At low particle density, we might be able to assume the presence of many single gold particles. In our experiments, following the second dewetting, we found that the number of plasmonic molecules with gaps less than 10 nm, which corresponds to the number of hotspots, increased (Figures S7–S9). In particular, it was confirmed that the 8 nm dual-dewetting substrate showed the largest SERS enhancement with a change in the number of hotspots. Analyzing the SEM images in Figure 3, it is obvious that the particles from the second dewetting are smaller than those from the single dewetting on the 8 nm dual-dewetting substrate. Using the average particle size values obtained from SEM, a plasmonic molecule composed of particles of different sizes was designed. The electric field distribution was checked for gold dimers, trimers, and tetramers to confirm the cases in which several small particles form respective hotspots around one large particle. In this case, compared to the single-particle case, we confirmed SERS enhancement from a few tens to hundreds of times at the hotspots, as shown in Figure 4b–d. The well-formed plasmonic molecules allowed us to predict SERS enhancement factors in hotspots from 200 to 500 times in dimers and trimers compared to single gold nanoparticles. In the tetramer, while the SERS enhancement is relatively low, it has a clearly enhanced value compared to the single gold particle case. By this point, it was confirmed that even when a large number of smaller particles form through the dual-dewetting process, superior SERS enhancement can be achieved compared to those by single particles.

**Figure 5:**
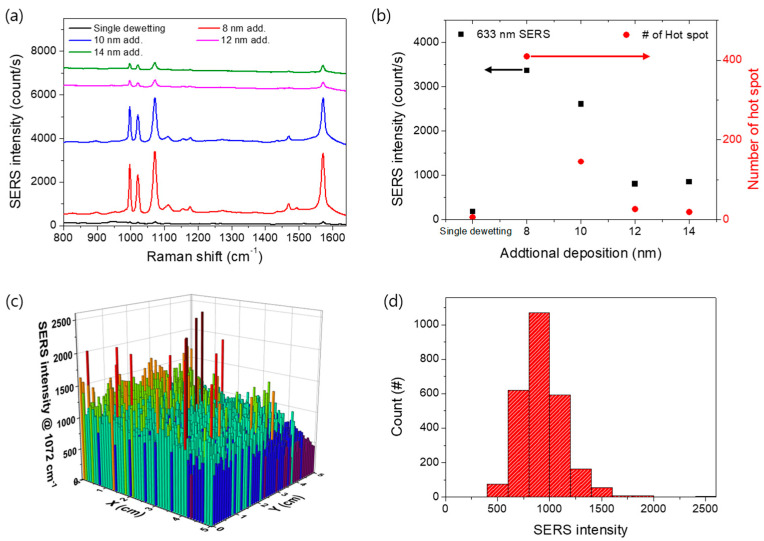
(**a**) SERS spectra of benzenethiol measured for each dewetting substrate. (**b**) SERS intensity of the 1072 cm^−1^ peak of benzenethiol (black squares) and number of hotspots (gaps below 10 nm) in a 4.8 µm^2^ area (red circles) as a function of additional deposition thickness and number of gaps smaller than 10 nm for each dewetting substrate. (**c**) Raman mapping data of a 5 cm × 5 cm large−area substrate subjected to dual−dewetting with 8 nm additional deposition at 1 mm intervals. (**d**) Histogram of SERS intensity from the mapping in (**c**).

SERS enhancement can be measured to check the improvement of plasmonic properties including electric field enhancement and hotspot distribution. To investigate the SERS performance, single and dual-dewetting substrates were coated with benzenethiol monolayer. Raman spectra were acquired utilizing a Raman spectrometer system (Horiba LabRAM HR-800) equipped with a 50× objective lens featuring a numerical aperture (NA) of 0.75 and a working distance of 0.38 mm. The excitation was provided by a 633 nm laser with an output power of 0.5 mW. The spectra were gathered over a 1 s accumulation period (Figure 5a). It can be seen that the unique Raman spectrum of benzenethiol, which was very weak in the single dewetting substrate case, comes out much more strongly from the dual-dewetting substrates.

To compare specific values of SERS enhancement, the SERS intensity of the 1072 cm^−1^ peak of benzenethiol under each dewetting condition was obtained, and the number of gaps smaller than 10 nm within a 4.8 µm^2^ area were counted (Figure 5b). As a result, it was confirmed that the 8 nm dual-dewetting substrate was shown to be 30 times stronger as an SERS enhancement than the single dewetting substrate. After a single dewetting, most gold nanoparticles failed to form plasmonic molecules, resulting in limited SERS enhancement. However, in the case of the dual-dewetting substrates, a significant number of plasmonic molecules with strong electric fields induced in sub-10 nm gaps were formed, leading to increased SERS enhancement. The 8 nm dual-dewetting substrate contained 409 sub-10 nm gaps within the 4.8 µm^2^ area, of which 238 were smaller than 5 nm with extremely large enhancement. Due to this large number of small gaps, the 8 nm dual-dewetting substrate exhibited the largest Raman enhancement among the dual-dewetting substrates. For the 10 nm dual-dewetting substrate, 145 gaps below 10 nm were found; otherwise, 26 and 19 such gaps were found for the 12 nm and 14 nm substrates, respectively. Although the substrate coverage increased for the 12 nm and 14 nm dual-dewetting substrates, on which plasmonic molecules were not well formed, these showed a relatively weak SERS enhancement of about 4.5 to 4.8 times compared to the single dewetting case (For dark-field spectra, see Supplement Figure S10).

To ensure substrate uniformity, Raman mapping was performed at 1 mm intervals on a large 5 cm × 5 cm area of a dual-dewetting substrate with additional 8 nm film deposition. To minimize the error due to the laser focus, mapping was performed using a 10× objective lens with a NA of 0.25 at an intensity of 1 mW (Figure 5c). The average SERS intensity was 980.4 counts with a deviation of about 21.4%, indicating that hotspots were evenly distributed across the substrate.

In this way, the dual-dewetting method showed the effect of increasing the number of plasmonic molecules over a large area in a very simple process, thereby enhancing the plasmonic properties. The number of hotspots was significantly increased by the greatly increased number of plasmonic molecules via dual-dewetting, and a large enhancement was confirmed from actual Raman signals. Accordingly, the proposed method is expected to be directly helpful for various plasmonic applications as well as SERS.

## 3. Materials and Methods

### 3.1. Dual-Dewetting Gold Nanoparticle Substrate Fabrication

A 6-inch Si wafer was coated with 300 nm of SiO_2_ through PECVD (TES TELIA 200) at a substrate temperature of 350 °C. Subsequently, 10 nm of gold was deposited onto the SiO_2_ layer using an E-beam evaporator (Korea Vacuum Tech, Gimpo, Korea, KVE-E2000L). The substrate was then heated to 820 °C in 90 s using rapid thermal annealing equipment (SNTEK, Suwon, Korea, RTP-5000) and maintained at this temperature for 2 min. After cooling, a gold thin film of the desired thickness was deposited using an E-beam evaporator. The same thermal process equipment was used to perform heat treatment under identical conditions as before. Structural confirmation was carried out using field emission SEM (Hitachi, Tokyo, Japan, S-4800) with an acceleration voltage of 10 kV and emission current of 10 µA.

### 3.2. Simulation

In this study, we utilized COMSOL Multiphysics (COMSOL Inc., Stockholm, Sweden, COMSOL 5.6), a commercial finite element method (FEM)-based simulation software, to explore the plasmonic properties of our structures. An x-polarized plane wave in the normal direction served as the incident light for estimating scattered light. Perfectly matched layers (PMLs) encased the simulated structures. A single set of plasmonic structures, such as a dimer, trimer, or tetramer, was employed in our simulations instead of periodic boundary conditions. Additionally, based on the SEM images, we shaped the gold particle geometry into 25% truncated spheres in diameter.

### 3.3. SERS Measurement

For SERS measurement, the nanogap substrate was immersed in an ethanol-diluted benzenethiol solution for over 48 h to allow self-assembly of the benzenethiol monolayer, followed by washing with pure ethanol. The SERS signal of benzenethiol was measured using a Raman spectrometer (Horiba, Kyoto, Japan, LabRAM HR-800) with a 50× objective lens having a NA of 0.75 and a working distance of 0.38 mm. A 633 nm laser was excited at a power of 0.5 mW. For SERS mapping, a 10× objective lens with a NA of 0.25 and a working distance of 10.6 mm was used, and the laser power was 1 mW.

## 4. Conclusions

We fabricated high-performance plasmonic molecules at high density through a dual-dewetting process without any lithography or chemical means. The proposed method provides a scalable approach that can be easily implemented on a 6-inch Si wafer with the expectation of equal implementation on larger substrates. Furthermore, by adjusting the thickness of the thin film for the second dewetting, the particle size distribution can be precisely controlled for various purposes. The increased number of particles from dual-dewetting increased the surface coverage 1.75 times, shortened the average interparticle distance, and dramatically increased the number of hotspots. This resulted in a 30-fold stronger enhancement of the SERS signal, compared to single dewetting, with an additional deposition of 8 nm film. The present research offers insights into designing plasmonic nanostructures with controllable size and density, providing a promising approach to advance the field of plasmonics and enable new applications with efficient arrangements of plasmonic molecules.

## Data Availability

Original data can be requested from the corresponding author.

## Supplementary Materials

The supporting information can be downloaded at: https://www.mdpi.com/article/10.3390/ijms241713102/s1.
