# Supplementary material for: Dual-Dewetting Process for Self-Assembled Nanoparticle Clusters in Wafer Scale
Source: Int J Mol Sci. 2023 Aug 23;24(17):13102. doi: 10.3390/ijms241713102 (PMC10488070; doi:10.3390/ijms241713102)
Supplement: Supplementary file 1 [file ijms-24-13102-s001.zip › ijms-2521954-supplementary.pdf]

Supplementary Information

Dual-Dewetting Process for Self-assembled Nanoparticle Clusters in Wafer Scale

Minjun Kim [1,†], Hyun-Ju Ahn [1,†], Vanna Chrismas Silalahi [1], Damun Heo [2], Samir Adhikari [1], Yudong Jang [4,*], Jongmin Lee [2,3,*] and Donghan Lee [1,4]

[1] Department of Physics, Chungnam National University, Daejeon 34134, Republic of Korea
[2] School of Semiconductor·Display Technology, Hallym University, Chuncheon 24252, Republic of Korea
[3] Nano Convergence Technology Cneter, Hallym University, Chuncheon 24252, Republic of Korea
[4] Institute of Quantum Systems (IQS), Chungnam National University, Daejeon 34134, Republic of Korea
* Correspondence: ydjang@cnu.ac.kr (Y.J.); jmlee@hallym.ac.kr (J.L.)
† These authors contributed equally to this work.

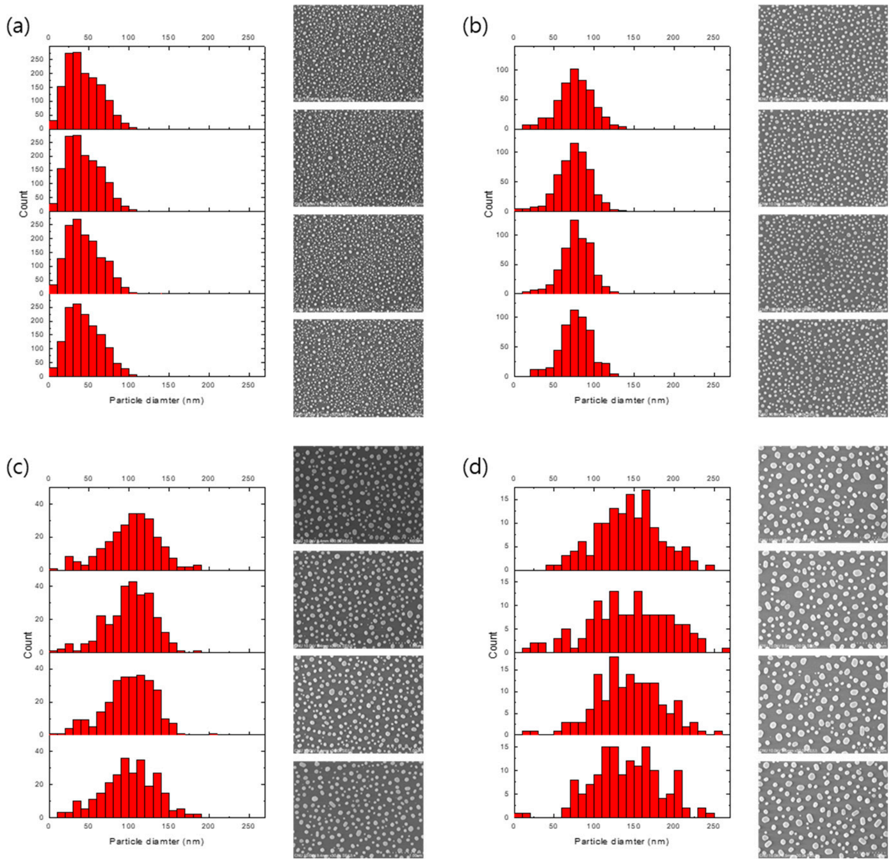

Figure S1. Particle distributions and corresponding SEM images after a single dewetting for different gold film thickness: (a) 8 nm, (b) 10 nm, (c) 12 nm, and (d) 14 nm.

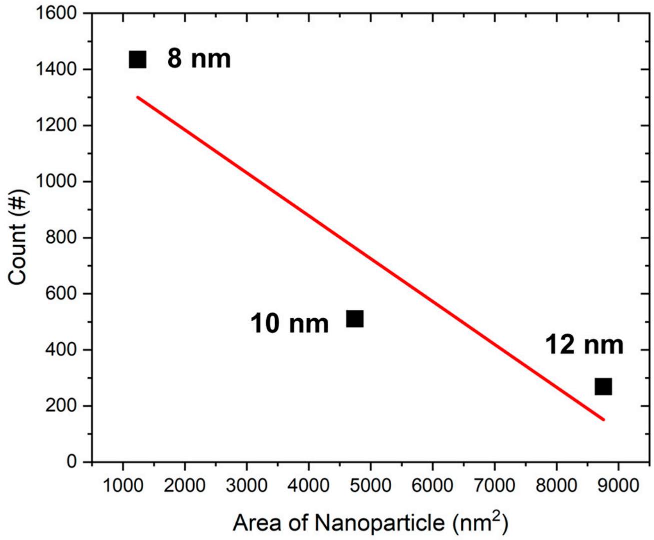

Figure S2. Graph of the correlation between nanoparticle size and number in a single dewetting process.

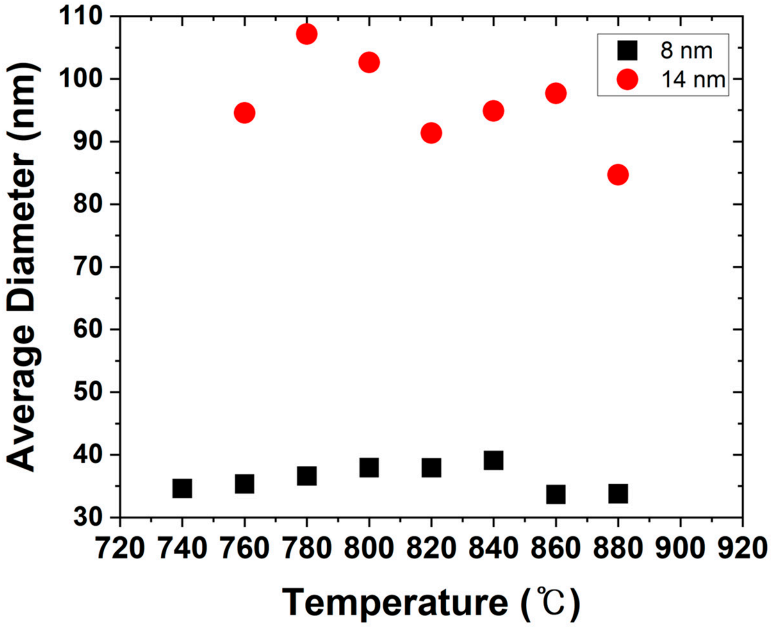

Figure S3. Average diameter of nanoparticles prepared from 8 nm and 14 nm gold films as a function of heat treatment temperature.

The nanoparticle diameters synthesized from 8 nm and 14 nm gold films were analyzed concerning the applied heat treatment temperatures. The 8 nm and 14 nm gold films were selected for investigation due to their representation of the extremities - the thinnest and thickest films, respectively, used in this study. The standard deviation pertaining to the average size of the nanoparticles fabricated with the 8 nm film was approximately 1.9 nm. However, there was a substantial increase in this figure to roughly 6.8 nm when the 14 nm film was utilized.

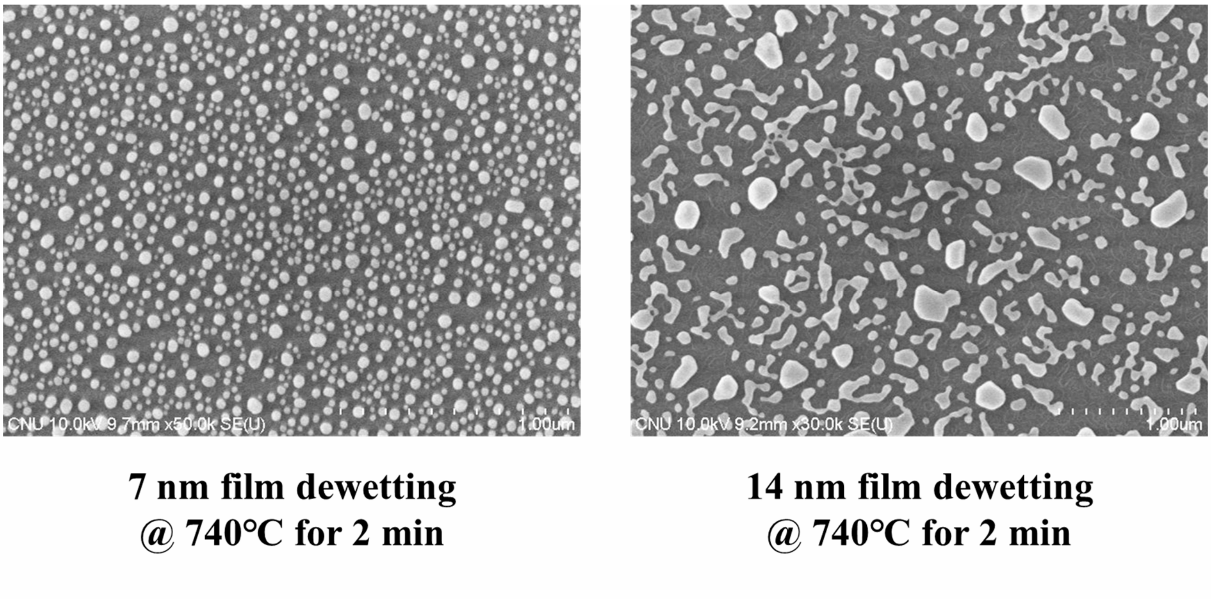

Figure S4. SEM images after a single dewetting with 7 nm gold film (left) and 14 nm (right).

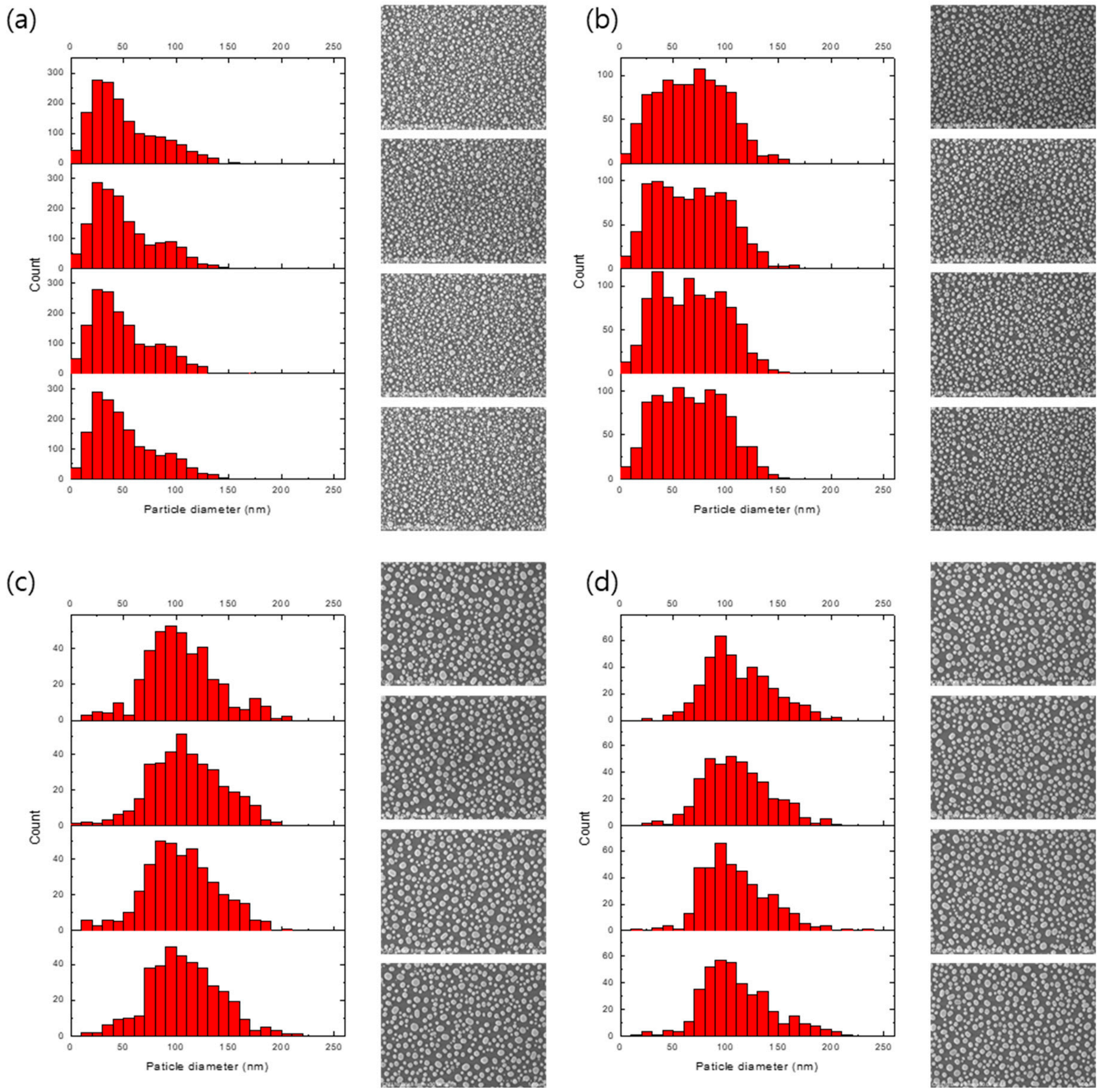

Figure S5. Particle distributions and corresponding SEM images after the second dewetting of a dewetted 10 nm thin film with additional deposition thicknesses of (a) 8 nm, (b) 10 nm, (c) 12 nm, and (d) 14 nm.

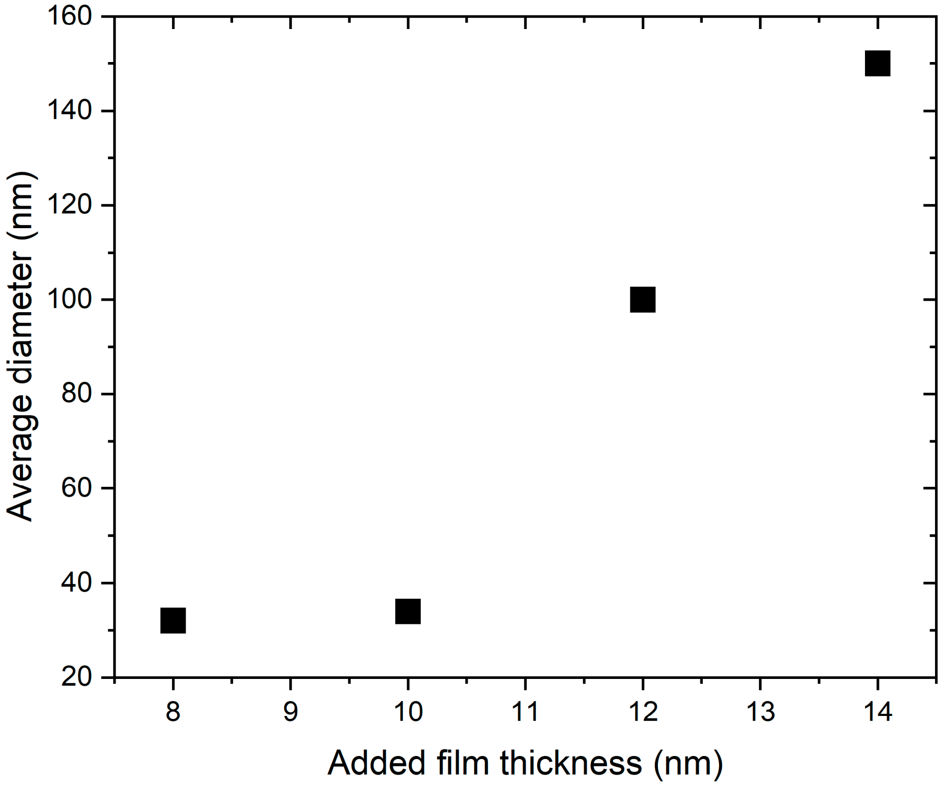

Figure S6. Average diameter of added nanoparticles by second dewetting as a function of added film thickness.

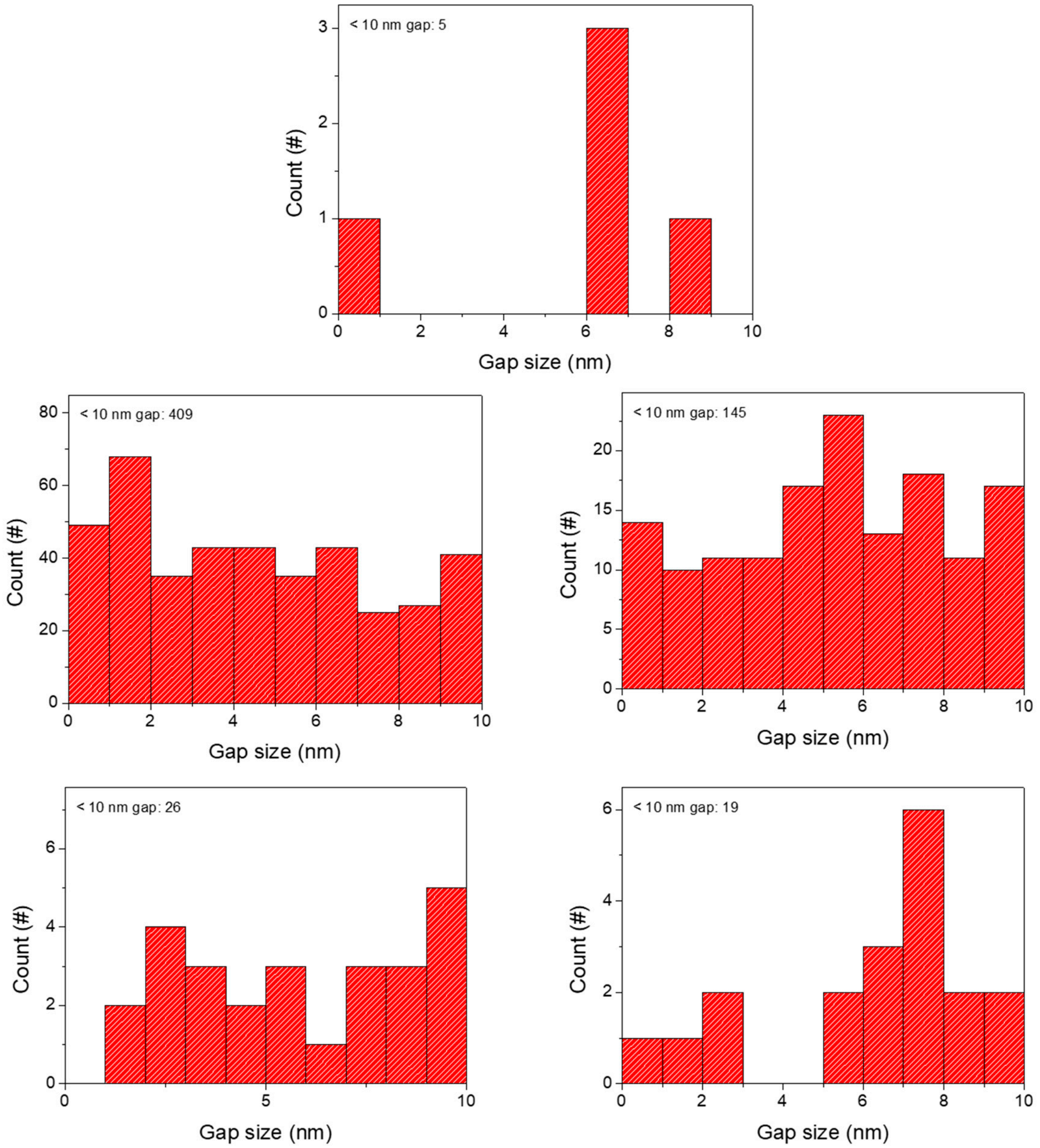

Figure S7. Distribution of gaps smaller than 10 nm within a 4.8 μm^2 area on a bare particle substrate, single dewetted 10 nm film, and dual-dewetting substrates with additional deposition.

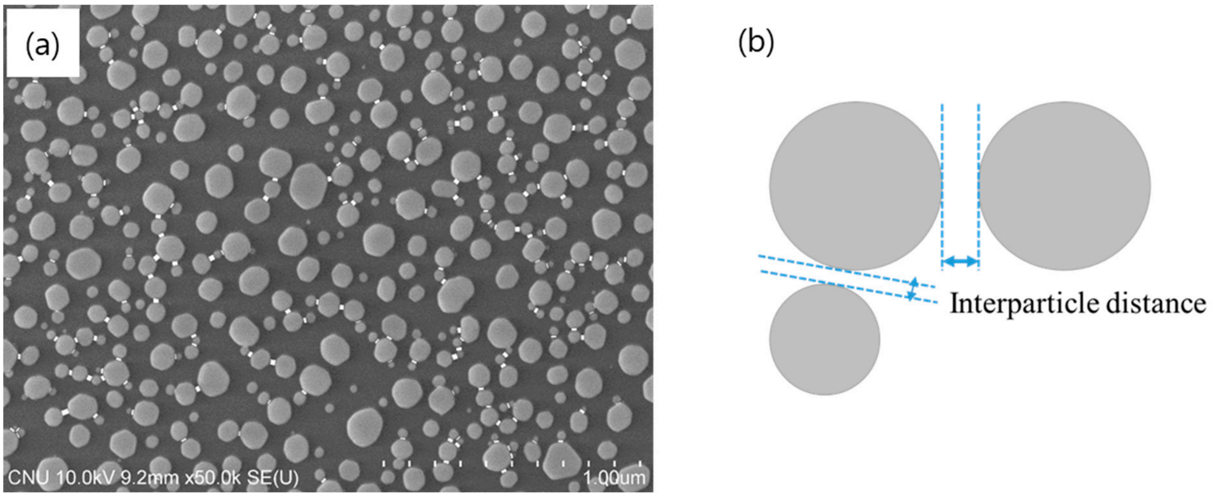

Figure S8. (a) SEM image of gold nanoparticles with interparticle distances labelled with the small white lines using ImageJ software. (b) Schematic of interparticle distance measurement between nanoparticles. The interparticle distances of gold nanoparticles were calculated using end-to-end distance measurements between different nanoparticles. Here, we have considered interparticle distances smaller than 10 nm only.

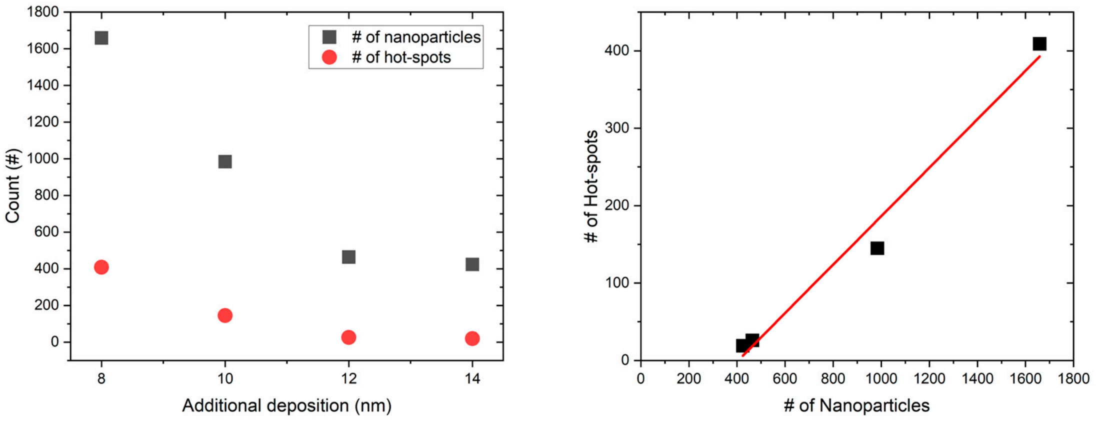

Figure S9. (Left) Additional deposited film thickness versus nanoparticle number (black dots) and hot-spot number (red dots). (Right) Correlation graph between nanoparticle number and hot-spot number.

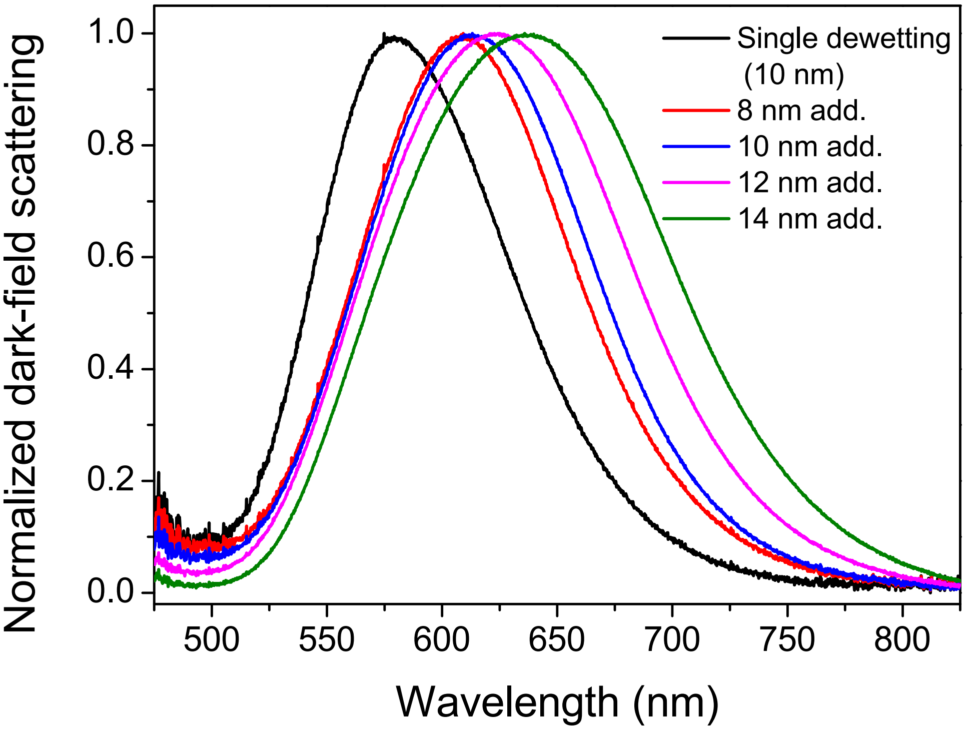

Figure S10. Normalized dark-field spectra of single dewetting and dual-dewetting substrates.

Supplementary Note 1. Relation between number of nanoparticles and number of hotspots

In order to elucidate the quantitative relationship between the quantity of nanoparticles and the number of hotspots, an examination of the characteristics of dewetted gold nanoparticles was conducted initially. The results of this investigative analysis are presented in Table S2, with the examination based on Supplementary Figure S1.

The study reveals that an increase in the initial film's thickness corresponds to an enlargement in nanoparticle size, concomitant with a decrease in nanoparticle number. As the formation of the nanoparticles is predicated on the confined resource of the gold film, there emerges an inverse proportionality between the increase in nanoparticle size and their numerical count (refer to Supplementary Figure S9).

Supplementary Figure S10 (left) delineates the number of nanoparticles (illustrated as black dots) and the number of hot spots (represented by red dots) in relation to the incremental thickness of the deposited film. This relationship between additional film thickness and the number of nanoparticles parallels the pattern observed with single dewetting-fabricated nanoparticles. Furthermore, a direct proportionality is discerned between the number of hotspots and the number of nanoparticles (Supplementary Fig. S10 (right)). Under the assumption that the nanoparticles are uniformly and randomly positioned within the plane during the secondary dewetting process, an elevated number of nanoparticles augments the likelihood of forming narrow interstitial gaps.

Table S1. Dimension of single dewetted gold particles

		Initial film thickness		
		8 nm	10 nm	12 nm
Gold Nanoparticles	Diameter (nm)	39.72 ± 0.7	77.73 ± 1	105.58 ± 1.74
	Area (nm^2)	1239.11	4745.34	8754.94
	Count (#)	1435.25 ± 19.75	511 ± 17.01	269 ± 11.25

Supplementary Note 2. Gaussian fit parameters of the particle size distribution following dual-dewetting

The Gaussian model used to check the distribution of the dewetted gold nanoparticles in Fig. 3 of the main text is as follows:

$$y = \frac{A}{\sigma\sqrt{2\pi}} e^{-\frac{1}{2}\left(\frac{x-\mu}{\sigma}\right)^2}$$

where *A* is the amplitude of the Gaussian curve, μ is the mean value, and σ is the standard deviation. The values used for each fitting are shown in Table S2.

Table S2. Parameters for the Gaussian fit model

		8 nm add.	10 nm add.	12 nm add.	14 nm add.	Single dewetting
Black	A	5100	6553	2215	1094	4855
	μ	80.6	82.0	86.7	91.7	75.6
	σ	25.9	25.1	19.1	19.9	38.5
Green	A	10950	3219	2785	3094	
	μ	32.5	34.0	102.4	144.2	
	σ	15.7	12.2	15.8	16.76	
